# Subcellular Drug Distribution: Exploring Organelle-Specific Characteristics for Enhanced Therapeutic Efficacy

**DOI:** 10.3390/pharmaceutics16091167

**Published:** 2024-09-04

**Authors:** Xin Liu, Miaomiao Li, Sukyung Woo

**Affiliations:** 1 Department of Pharmaceutical Sciences, School of Pharmacy and Pharmaceutical Sciences, State University of New York at Buffalo, Buffalo, NY 14214-8033, USA; liuxinliaa@gmail.com; 2 Division of Biosciences, College of Dentistry, The Ohio State University, Columbus, OH 43210-1267, USA; li.15541@osu.edu

**Keywords:** subcellular drug distribution, drug resistance, drug efficacy, organelle targeting, computational pharmacokinetic modeling

## Abstract

The efficacy and potential toxicity of drug treatments depends on the drug concentration at its site of action, intricately linked to its distribution within diverse organelles of mammalian cells. These organelles, including the nucleus, endosome, lysosome, mitochondria, endoplasmic reticulum, Golgi apparatus, lipid droplets, exosomes, and membrane-less structures, create distinct sub-compartments within the cell, each with unique biological features. Certain structures within these sub-compartments possess the ability to selectively accumulate or exclude drugs based on their physicochemical attributes, directly impacting drug efficacy. Under pathological conditions, such as cancer, many cells undergo dynamic alterations in subcellular organelles, leading to changes in the active concentration of drugs. A mechanistic and quantitative understanding of how organelle characteristics and abundance alter drug partition coefficients is crucial. This review explores biological factors and physicochemical properties influencing subcellular drug distribution, alongside strategies for modulation to enhance efficacy. Additionally, we discuss physiologically based computational models for subcellular drug distribution, providing a quantifiable means to simulate and predict drug distribution at the subcellular level, with the potential to optimize drug development strategies.

## 1. Introduction

Once in circulation, the targeting strategy first requires drugs to traverse the plasma membrane. This can occur through passive diffusion for small, lipophilic drugs, active transport via specific transporter proteins for hydrophilic or larger molecules, endocytosis for certain biologics and nanoparticles, membrane permeabilization, or receptor-mediated uptake. These entry mechanisms are essential for understanding and optimizing drug distribution and have been relatively well studied. Consequently, the efficacy of treatment and its potential toxicity are determined by the concentration of the drug at its site of action, which is closely tied to the drug’s distribution within the cell’s subcellular structures. Mammalian cells contain a variety of organelles, including the nucleus, lysosome, mitochondria, endoplasmic reticulum (ER), lipid droplets, Golgi apparatus, and other membrane-less structures [1], such as liquid–liquid phase separation. These distinct structures possess unique biological characteristics and functions, effectively dividing the cell into multiple sub-compartments, each of which may exhibit distinct rates and extents of drug partitioning. As a result, drug concentrations within the cell are not homogenous, and this sub-compartmentalization provides a more accurate description of drug distribution within the cell, facilitating the calculation of drug concentration at the site of action.

Within these sub-compartments, certain structures have the capacity to selectively accumulate or exclude drugs based on their physicochemical attributes. This selectivity has a direct impact on drug efficacy by shaping their distribution within these compartments. The process of drug distribution is influenced by various physiological factors, including membrane potential, pH gradients across membranes, lipid composition, and the expression levels of drug transporters. Key characteristics such as lipophilicity, size, and charge play a role in determining the distribution pattern of drugs.

To comprehend the intricate relationship between cellular sub-compartments and intracellular drug distribution, it is important to consider the relative abundance of specific organelles in target tissues. Under pathological conditions, such as cancer, many cells undergo dynamic alterations in subcellular organelles. For instance, lung tissue is enriched in lysosomes, while mitochondria are abundant in cardiac muscle cells. Certain cancer cells even accumulate lipids to ensure the energy production and membrane synthesis required for their rapid growth. Moreover, it is critical to attain a mechanistic and quantitative understanding of how the organelle characteristics alter the partition coefficient of drugs among them.

This comprehensive review aims to provide insights into the intricate interplay between subcellular distribution and drug efficacy, shedding light on how the abundance and characteristics of cellular organelles significantly impact the overall therapeutic outcome. The review also delves into the key biological factors and physicochemical properties that contribute to specific subcellular drug distribution, along with strategies employed to modulate such distribution for improved efficacy. Lastly, it discusses physiologically based computational models for subcellular drug distribution, illustrating an approach that offers quantifiable means to simulate and predict drug distribution at the subcellular level. This computational model holds the potential to support strategies aimed at enhancing the efficiency of drug development and optimization.

## 2. Lysosome

### 2.1. pH Gradient Drives Accumulation and Drug Resistance

Lysosome, an acidic organelle, plays an important role in cellular digestion. It has implications for cancer because it can retain chemotherapeutic drugs in its cationic state. This retention of drugs can diminish their cytotoxic effectiveness by decreasing the drug concentration at the active site, ultimately resulting in multidrug resistance.

To date, approximately 25% of anticancer chemotherapeutic agents are bases/weak bases. The quantification of this characteristic is often carried out using the pKa value, where a basic drug is classified as having a pKa greater than 6.0 [2]. These types of drugs tend to ionize in environments with low pH, and the ionization tendency increases with a higher pKa value. Consequently, this ionization makes it challenging for the drugs to diffuse across biological membranes, leading to their accumulation in lysosomes.

Research by Gotink et al. [3] demonstrated that the accumulation of sunitinib within lysosomes significantly contributes to the development of drug resistance. Sunitinib, a weak basic drug (pKa value of 8.95), exhibits clear colocalization with lysosomes. Since sunitinib is mainly metabolized by CYP3A4 in the liver, minimal metabolism occurs in other tissues, including cancer cells. As a result, sunitinib stored in lysosomes can eventually re-enter the cytosol and bloodstream. However, since the ratio of drug concentration between lysosomes and the cytosol remains constant, lysosomal sequestration of sunitinib reduces its availability in the cytoplasm—where its primary targets, VEGFR2 and PDGFR, are located—despite high overall drug levels. This diminished cytoplasmic availability lowers its efficacy and contributes to the development of drug resistance. Additional studies have also confirmed increased lysosomal sequestration in sunitinib-resistant cells [4,5,6,7,8]. Moreover, Faraz et al. [2] revealed that lapatinib and gefitinib are susceptible to being rendered ineffective due to lysosomal sequestration. Similar to sunitinib, both lapatinib and gefitinib possess comparable physicochemical attributes, including high logP values (5.4 and 3.2, respectively), facilitating their efficient passage through the cell membrane. Their weak basic characteristics (pKa 7.26 and 6.83) contribute to lysosomal accumulation, impacting their therapeutic effectiveness.

Numerous studies strongly support the concept of ionization-induced lysosomal sequestration as a mechanism of drug resistance. Drugs like doxorubicin, mitoxantrone, vincristine, sorafenib, nintedanib, and imatinib have all been reported to exhibit this phenomenon [4,9,10,11]. Understanding this mechanism opens new avenues for developing strategies that can overcome drug resistance and improve the outcome of cancer therapies.

### 2.2. Regulation of pH Gradient

Cellular sub-compartments maintain a stable pH through the delicate balance of proton pumping and counterion conductance In cancer cells, this equilibrium is often disrupted, leading to an acidic cytoplasmic pH and a reduced pH gradient between lysosomes and the cytoplasm [12]. Interestingly, this characteristic renders tumors more responsive to basic drugs, as more drug remains in the cytosol. When exposed to chemotherapeutic treatments, cells attempt to restore pH balance by alkalinizing the cytoplasm to elevate the pH gradient with lysosomes [13].

The phenomenon of altered pH levels has been extensively studied in the MCF7 cell line. These cells typically exhibit a lysosome/cytosol pH of 5.8/6.8, lower than that of normal cells (5.0/7.2). Doxorubicin treatment elevates these pH values to 5.5/7.1 [14,15]. While agents specifically designed to directly modulate lysosome pH are limited, disrupting lysosomal sequestration remains a viable strategy to reverse drug resistance. Proton-pumping vacuolar-ATPase proteins play a major role in regulating lysosome pH. Inhibitors of these proteins, such as bafilomycin A1 and concanamycin A, can abolish the pH gradient, releasing agents from lysosomes to their intended sites of action [15]. Qiao et al. [16] highlighted that the high expression of KCNJ15 protein in cancer cells which can reduce the PH gradient of lysosome through disrupting V-ATPase function leads to restore drug efficacy. Basic drugs like chloroquine and hydroxychloroquine, along with small molecules like NH_4_Cl can accumulate in lysosomes and elevate pH levels [17,18]. Understanding the interplay between pH regulation and drug sensitivity provides insights for developing novel approaches to enhance cancer treatment effects.

### 2.3. Transporters Facilitate Accumulation

Duvvuri et al. [19] made an interesting observation regarding certain basic compounds. Their findings indicate that the lysosomal concentration of these compounds exceeds predictions based on pH-partitioning alone. Experimentally determined values were approximately 3−15 times higher than theoretically predicted values, suggesting that active transporters, such as ATP-binding cassette transporter (ABC) system, potentially play a role in driving drug accumulation within the lysosome. Studies have revealed the involvement of P-glycoprotein, localized on the lysosomal membrane, in the translocation of doxorubicin from the cytosol to the lysosomes [20,21]. This mechanism prevents doxorubicin from interacting with molecular targets in the cytosol, leading to a reduction in its effectiveness. Moreover, Chapuy et al. [22] have shown that overexpression of the intracellular ATP-binding cassette transporter A3 (ABCA3) contributes to daunorubicin resistance in cancer cells. ABCA3 enhances the sequestration of the drug in lysosomes, further limiting its availability in the cytosol and subsequently promoting drug resistance. In addition, Herman et al. [23] identified SLC22A1 as a transporter capable of transporting imatinib to lysosomes. Notably, the sequestration process is not solely dependent on SLC22A1 expression, as additional factors like pH, temperature and energy also contribute to imatinib’s accumulation within lysosomes.

### 2.4. Lysosome as a Target for Anti-Cancer Therapies

While lysosomes play a role in multidrug resistance by sequestering basic drugs, they also present a promising target for anticancer therapies. Lysosomal membrane permeabilization, when it occurs, has the potential to turn lysosomes into lethal organelles that release molecules like cathepsin into the cytosol, ultimately triggering apoptosis [24]. An intriguing discovery by Ikuko et al. [25] demonstrates this potential, as L-Leucyl-L-leucine methyl ester (Leu-Leu-OMe) induces cell death under autophagy-suppressed conditions. Leu-Leu-OMe is converted to a detergent within lysosomes by cathepsin C, which disrupts the lysosomal membrane [26]. Additionally, the identification of polyphyllin D as a lysosome-targeted compound causing lysosomal damage through a lysosometropic-based in silico screening by Wang et al. [27] highlights similar possibilities. Agents like ciprofloxacin, sphingosine, and siramesine have also demonstrated lysosomal membrane damaging effects [28,29,30]. Furthermore, both in vitro and in vivo studies have reported that tumor necrosis factor alpha (TNFα) can induce lysosome permeabilization [31,32,33]. Another source of lysosomal damage is reactive oxygen species (ROS), which can cause membrane peroxidation and disrupt proteins attached to lysosomes. ROS production can occur due to oxidative stress, inflammation, and an excess of hydrogen peroxide within lysosomes [34]. These studies further support the potential significance of lysosomes in therapeutic strategies.

## 3. Mitochondria

The mitochondrion is a crucial sub-compartment within cells, responsible for vital functions such as energy generation, material metabolism, and activation of signaling pathways [35]. Compounds prone to accumulating in mitochondria possess specific characteristics that enhance their absorption and extended presence within these cellular organelles. Lipophilic properties facilitate passive diffusion through the lipid-rich mitochondrial membranes, while positive charges are electrostatically attracted to the negatively charged environment. Moreover, the utilization of mitochondrial targeting peptides facilitates active transport, ensuring efficient and selective accumulation. Factors like size, shape, binding affinity for mitochondrial components, chemical stability, responsiveness to ROS and pH gradients, and active transport capacity further influence the mitochondrial uptake capacity. By harnessing these characteristics, researchers can design compounds that preferentially accumulate within mitochondria, presenting opportunities for targeted therapeutics in mitochondrial-related conditions.

### 3.1. Membrane Potential Drives Accumulation in Mitochondria

Mitochondria possess distinct features that set them apart from other organelles. Comprising an outer membrane (OMM) with porins and an inner membrane (IMM), the abundance of porins in the OMM facilitates easy passage of compounds. However, the IMM is nonporous, and has a rich content of cardiolipin in the bilayer phospholipids, rendering it impermeable to most molecules. Additionally, a membrane electrochemical gradient exists between the mitochondrial matrix and intermembrane space, leading to an alkaline intra-organelle matrix with a pH of 8.0 (slightly higher in tumor cells). This unique feature attracts molecules with positive charges [36].

Some molecules with high hydrophobicity and positive charges, like triphenylphosphonium (TPP), have been investigated and found to pass through the IMM and accumulate in the mitochondrial matrix [37]. Min et al. [38] conjugated doxorubicin (DOX) with TPP for selectively delivering the complex to mitochondrion. This complex significantly increased the concentration of DOX in drug-resistant cell lines, enhancing its cytotoxicity and partially overcoming drug resistance. Curcumin, known for inducing apoptosis in cancer cells by activating caspase 3 and releasing cytochrome c in mitochondria, has limited clinical benefits due to its low bioavailability in the bloodstream and tissues. Cheruku et al. [39] developed curcumin-TPP conjugates that resulted in significantly higher efficacy than free curcumin in multiple cancer cell lines. Other moieties, such as phenylsulfonyl furoxan, Gboxin, and rhodamine were also used for conjugation to drug targeting delivery. Notably, even molecules lacking lipophilicity and positive charges can accumulate in mitochondria. An example is poly (2-Noxide-N,N-diethylamino)ethyl methacrylate (OPDEA) [40] found highly localized in mitochondria, which is zwitterionic with a zeta-potential of about 0.95 mV in deionized water. It was conjugated with dichloroacetate (DCA) [41] and the complex inhibited pyruvate dehydrogenase kinase 1 which initiates the mitochondrial oxidative stress and subsequent tumor cell apoptosis.

### 3.2. Mitochondria-Targeting Peptides

Apart from utilizing mitochondrial membrane potential as a driving strategy for mitochondrial targeting, another approach involves exploiting the protein import machinery engaged in mitochondrial formation. By conjugating chemotherapeutic agents with sequences that facilitate protein translocation to the mitochondrial membrane during mitochondrial biogenesis, mitochondrial targeting has been explored. For instance, researchers have linked mitochondrial-penetrating peptide (MPP) to the chemotherapeutic agent DOX and shown that MPP-DOX can be effectively distributed to tumors and overcome drug resistance both in vitro and in vivo [42]. Additionally, certain artificial peptides with similar functions have been developed. Young et al. [43] designed a cell-penetrating artificial mitochondria-targeting peptide (CAMP) to facilitate the transduction of the antioxidant protein human metallothionein 1A (hMT1A) into mitochondria, enhancing the effect of hMT1A by increasing tyrosine hydroxylase expression. Surprisingly, the protective role of mitochondria has been observed in other contexts. For example, with methotrexate, an antimicrobial agent that inhibits dihydrofolate reductase (DHFR) activity, Mark et al. [44] demonstrated that mitochondria could trap peptide-conjugated methotrexate, reducing its toxicity to host cells. This alteration in subcellular distribution enables methotrexate to retain its activity against various pathogenic Gram-positive organisms while minimizing harm to host cells. These studies underscore the potential of designing mitochondrial-targeted complexes to enhance the efficacy of anticancer agents and address drug resistance, offering promising approaches for improving cancer treatment outcomes.

### 3.3. Mitochondria as a Target for Anti-Cancer Treatment

Due to its central role in numerous essential cellular processes, targeting specific compounds to accumulate within the mitochondria can have therapeutic applications, especially in the context of diseases where mitochondrial dysfunction plays a role, such as cancer. Furthermore, mutations in mitochondria cause deregulation of cell viability signaling and lead to cancer progression and chemoresistance [45,46]. Various strategies have been explored to target mitochondria in cancer treatment. These strategies include reducing ATP production, inducing mitochondria outer membrane permeation, inhibiting the respiratory pathway, and inducing damage to mitochondrial DNA (mtDNA). Each of these approaches aims to disrupt the integrity and function of mitochondria, which could potentially lead to the selective elimination of cancer cells and enhance the effectiveness of chemotherapy against tumors [47,48].

The aforementioned strategies involve using different anticancer agents, each capable of killing tumors through various mechanisms. Bedaquiline, which inhibits mitochondrial ATP-synthase through downregulating ATP5F1C expression, can selectively prevent cancer growth and metastasis, without including normal cell death in cells with relatively low ATP-production rate [49]. Cheng et al. [50] developed a self-delivery chimeric peptide-based nanoparticle (M-ChiP) for synergistic anti-cancer therapy, which enhances mitochondrial membrane permeability and generation of ROS, initiating cancer cell apoptosis and necrosis. Jiang et al. [51] developed a novel Fe^2+^–Ru^2+^-loaded mesoporous silica nanoparticle to induce oxidative damage in the mtDNA of tumor cells. The oxidative mtDNA can activate macrophages against tumor cells by binding with M1-polarize tumor-associated macrophages (TAMs) after escaping from cancer cells.

However, as treatments progress, challenges like acquired drug resistance and toxic side effects often arise. To address these issues, a drug-free strategy has emerged, involving the development of specific complexes. These complexes do not exhibit toxic effects on cancer cells but can target mitochondria and assemble to create unique structures that physically block and interrupt mitochondrial function [52].

One such approach involves the use of trialkoxysilane, which induces silicification through hydrolysis and condensation. This damages the organelle membrane and generates an excess of ROS. In experiments conducted by Kim et al. [53], the trialkoxysilane-TPP combination was tested on cancer cells and tumor-bearing mice. Silicification was observed solely in SCC7 cancer cells but not normal cells, as their mitochondria have a higher pH value, allowing them to trap more trialkoxysilane. In a mouse model, the combination led to tumor growth inhibition, while kidney cells (HEK293T) remained unaffected. Another approach by Zhao et al. [54] involves a Z-scheme SnS1.68-WO2.41 nanocatalyst that generates holes and hydrogen molecules in mitochondria under near-infrared catalysis. Combined hole/hydrogen can depress cancer cell energy metabolism, enhance intratumoral ROS levels and induce DNA damage. Importantly, the nanocatalyst remains unharmed during the healing process, maximizing its therapeutic potential. Zhao et al. [55] reported the use of folate receptors that are more abundant in cancer cells compared to normal cells. Folic acid accumulation occurs as a result, leading to increased calcium attraction and mineralization which initiates the formation of a solid layer that covers the cell surface, blocking communication with the extracellular matrix and ultimately killing the cancer cells.

## 4. Endoplasmic Reticulum (ER)

The ER is a crucial organelle responsible for protein synthesis, folding, modification, and transport, as well as lipid synthesis and calcium storage. It extensively communicates with other organelles to maintain cellular homeostasis and coordinate various physiological processes. The ER plays a pivotal role in disease biology, influencing cellular metabolism, stress responses, and therapeutic outcomes. Understanding the functions of the ER can inform the development of targeted therapies.

### 4.1. ER Stress and Drug Resistance

ER stress (ERS) is a condition that arises in response to stresses such as nutrient depletion, hypoxia, oxidative stress, or chemotherapies, which disrupt the function or structure of the ER, leading to the accumulation of unfolded or misfolded proteins. Moderate ERS can be restored, but excessive stress may trigger apoptosis. ERS activates a cellular response known as the unfolded protein response (UPR), a mechanism aimed at restoring normal ER function to protect cells. However, UPR can also contribute to drug resistance. ERS triggers several signaling pathways associated with cell survival, helping cells cope with stress. GRP78 is a chaperone in the ER that manages protein quality by regulating folding and assembly and promotes epithelial–mesenchymal transition (EMT) and the stemness of cancer cells. Overexpression of GRP78 has been observed in various cancers, resulting in drug resistance [56,57,58]. Other proteins, such as PERK, IRE1α, and ATF6, are also activated and overexpressed under ERS, initiating UPR signaling cascades that consequently regulate protein transcription, synthesis, folding, secretion, and degradation. These processes reduce drug-induced apoptosis in tumor cells, contributing to drug resistance [59,60,61,62]. Additionally, ABC transporters play a key role in causing drug resistance. Gao et al. [63] reported that the anticancer drug 5-fluorouracil (5-FU) activates the URP and IRE1α-XBP1 pathway, inducing the expression of ABCB1, ABCC1 and ABCG2 in colon cancer cells, and inhibiting IRE1α RNase can restore drug sensitivity.

### 4.2. ER as a Target for Anticancer Treatment

Targeting the ER in cancer therapy offers significant potential by disrupting protein synthesis, calcium signaling, and stress responses, thereby impeding cancer cell survival and growth. Various strategies have been developed to deliver drugs to the ER, with small molecules being a common option for drug conjugation. Glibenclamide and its derivatives are widely used due to their high affinity and specific selectivity towards the ER-abundant sulfonamide receptor. Zhou et al. [64] synthesized two novel boron-dipyrromethene (BODIPY)-based photosensitizers with a structure similar to glibenclamide, achieving IC_50_ values as low as 0.09 μM due to their ER specificity. He et al. [65] developed another BODIPY-based probe for ER targeting, conjugated with molecules that bind to the chloride pump on the ER, capable of monitoring the real-time dynamics of biothiols.

In addition to small molecules, specific peptides are also effective carriers. Pardaxin, a natural cationic peptide derived from fish, was shown by Ting et al. [66] to specifically traffic to the ER through a non-lysosomal pathway, inducing activator protein-1 expression and resulting in apoptosis of HT-1080 cells. Synthesized peptides known as ER resident signal peptides (ERSPs) have been developed for ER targeting, with modifications to track location and evaluate drug effectiveness [67].

Recently, the interplay between the ER and mitochondria has gained attention due to its critical role in lipid and ion transfer, metabolism, autophagy, and other processes, making the ER–mitochondria contact site a strategic target. Li et al. [68] revealed that in cisplatin-resistance ovarian cancer, the formation of Mitochondria-associated ER Membrane (MAM) was enriched by the GRP75 pathway, facilitating Ca^2^⁺ fluxes crucial for tumor survival under stress. Disrupting MAM formation is thus important for restoring drug effectiveness in resistant cells. Ciscato et al. [61] reported that using a selective peptide to displace isoform 2 of the glycolytic enzyme hexokinase (HK2) located on MAM induces mitochondrial depolarization and apoptosis due to the Ca^+^ overload in the mitochondrial, highlighting MAM as a potential anticancer target. Overall, these studies demonstrate the potential of targeting the ER or MAM for cancer treatment, underscoring their promise as therapeutic targets.

## 5. Lipid Droplets

Lipid droplets (LDs) are organelles prevalent in most eukaryotic cells, responsible for storing and metabolizing neutral lipids, such as triglycerides and cholesterol esters. They serve as platforms for various metabolic enzymes involved in lipid biosynthesis and degradation processes. Numerous lipid-related proteins are present on the surface of LDs’ monolayer membrane, playing a crucial role in facilitating extensive communication between LDs and other organelles. Through contact sites, materials can be transferred between LDs and organelles, regulating lipid metabolism, organelle biogenesis, autophagy, and other stress responses [69,70,71,72].

As researchers discover the increasing significance of LDs in cellular activities, they have also begun exploring the relationship between LDs and cancer. In cancer cells (such as breast, colon, and lung cancer), there is a tendency to uptake more lipids [73,74] and enhance de novo lipid synthesis to form more LDs through expressing more fatty acid synthase (e.g., acetyl-CoA synthetases (ACSS), Stearoyl-CoA Desaturase 1 (SCD1), ADRP/Perilipin 2, and ATP citrate lyase (ACLY)) [75,76,77,78,79]. These alterations provide cancer cells with a greater capacity to survive in different stressful environments and contribute to tumor aggressiveness [80]. A new study conducted by Zhao et al. [81] revealed that cells can resist ROS and lipotoxicity via uptaking perilipin-coated artificial lipid droplets.

### 5.1. Drug Accumulation in LDs and Drug Resistance

Numerous investigations have revealed a positive correlation between drug resistance and LD content, where drug-resistant cells exhibit higher LD levels compared to drug-sensitive cells [82,83,84]. In FGFRI-driven lung cancer cells, Berhard et al. [85] discovered that resistance to the drug ponatinib is associated with elevated LD formation. Ponatinib, being a highly lipophilic compound with a logP value of 4.32, can accumulate within LDs, acting as a reservoir that reduces the available drug concentration at its site of action. Confocal colocalization results supported the notion that ponatinib becomes arrested in LDs. Similar results were reported by Quanfu et al. [82], where the drug gefitinib, possessing a high logP value (3.2), showed reduced cytotoxic effects in cells with higher LD contents. Oleic acid also diminished gefitinib’s efficacy by inducing LD formation in gefitinib-sensitive cells. Conversely, when LD levels were reduced by inhibiting SCD1 expression, drug resistance could be reversed in both in vitro and in vivo experiments. Summarizing these studies, ponatinib and gefitinib, being tyrosine kinase inhibitors (TKIs) with high logP values, have their sites of action in the cytoplasm. LDs can sequester a significant portion of these compounds, affecting their subcellular distribution and reducing their cytosolic concentration.

It is possible that more TKI drugs with similar characteristics may be found, but it is important to note that LD accumulation is not solely governed by logP values. Factors such as pKa, pH of sub-compartments, and transporters also play important roles in subcellular drug distribution. Furthermore, the accumulation of drugs in LDs does not always lead to drug resistance; it depends on the drug’s target sites. For example, lasonolide A, a polyketide-derived macrolide, can accumulate in lipid droplets. However, its target, lipid droplet-associated hydrolase (LDAH), is located on the surface of lipid droplets. Unlike TKIs, whose targets are in the cytoplasm, the accumulation of lasonolide A enhances drug activation and promotes cancer cell death [86].

### 5.2. LDs as a Target for Anticancer Treatment

Chemotherapeutic agents can induce ER stress, leading to apoptosis in cancer cells. However, in response to these changes, LDs, which are ER-derived organelles, tend to form and contribute to drug resistance. Therefore, targeting LD formation may represent a potential therapy to overcome drug resistance when combined with traditional chemotherapy drugs.

Issan et al. [87], reported that pyrrolidine-2, a cytoplasmic phospholipase A2 alpha inhibitor, sensitized glioblastoma multiforme to curcumin by reducing LD number and preventing drug sequestration in them, both in 2D and 3D experiments. Similarly, inhibitors of SCD1 (g-PPT) and FAS (orlistat) were found to impair lipid synthesis, thereby reducing LD levels in cancer cells, and enhancing the efficacy of combined drug treatments [82,88,89]. Li et al. [90] constructed a novel compound DPP-BPYS which can target LDs in tumor cells and produce ROS to induce apoptosis with its H_2_O_2_-activated form. SSR-LDs is another LD-targeted fluorescent probe that can serve as a tool for LD-related disease diagnosis and drug delivery [91]. Other research has demonstrated that inhibiting lipid catabolism can sensitize cancer cells and induce apoptosis. The CPT1 inhibitor, etomoxir, was used in lung, prostate cancer, and leukemia cells to reduce lipolysis and enhance drug sensitivity [89,92,93].

## 6. Nucleus

The nucleus is a central organelle in eukaryotic cells, housing the majority of the cell’s DNA and serving as the platform for controlling genetic information storage, replication, and transcription. As a result, it plays a vital role in regulating cellular processes and progression. In cancer therapy, numerous drugs have been developed to target the nucleus and interfere with gene expression. The nucleus is enclosed by double-layered membranes and traversed by nuclear pore complexes (NPCs), serving as the main channel for biomolecule transport. Unlike lysosomes or mitochondria, the nucleus relies on NPCs employing diffusion and active translocation mechanisms, rather than pH gradient or membrane potential, as the critical driving force for drug transport.

### 6.1. Nucleus Entry of Molecules through NPCs

The NPCs have a mean diameter of only about 9 nm, which restricts the entry of large molecules from the cytoplasmic compartment [94,95,96]. The efficiency of passive diffusion depends on the size and shape of the drug complex. Huo et al. [97] investigated the penetration ability of gold nanoparticles of different sizes for intranuclear delivery and found that nanoparticles with diameters > 10 nm cannot penetrate the nucleus, while smaller nanoparticles can enter the nucleus. Additionally, triplex-forming oligonucleotides (TFO) conjugated with small nanoparticles were more effective in reducing c-myc mRNA levels compared to free TFO. Liping et al. [98] developed a size-photocontrollable nanodrug delivery system to overcome multidrug resistance caused by P-gp overexpression in cancer cells. The DOX-containing nanodrug with a diameter < 10 nm can enter the nucleus through NPCs, restoring sensitivity in drug-resistant cancer cells. Furthermore, Hinde et al. [99] discovered that nanoparticle shape can also influence translocation to the nucleus. They tested nanoparticles with identical surface areas but varying shapes, including micelles, vesicles, rods, and worms. Despite micelle and vesicle shapes having a greater diameter than 10 nm, preventing them from passing through NPCs, rod and worm nanoparticles have long lengths and diameters under 10 nm, making it possible for them to diffuse into the nucleus. In summary, the size and shape of the drug-delivering complex play a crucial role in their ability to efficiently reach the nucleus through NPCs.

In addition to noninvasive strategies for nuclear targeting, researchers have devised a method based on modulating the nuclear membrane and enlarging NPCs on its surface to facilitate the entry of large molecules into the nucleus. Zhu et al. [100] developed a nanoparticle platform consisting of a polyhedral oligomeric silsesquioxane unit, a hydrophilic polyethylene glycol chain, and the photosensitizer Rose Bengal, that can localize within lysosomes and disrupt their function by generating singlet oxygen through oxidation. This disruption, in turn, impacts the nuclear membrane structure, promoting the entry of drugs into the nucleus. Notably, this approach allows large-sized compounds to enter the nucleus without requiring complicated modifications with a nuclear localization signal.

### 6.2. Active Translocation Involves Nuclear Localization Signal (NLS)

The diffusion of nanoparticles through NPCs is affected by the size of these complexes. Active transport processes, on the other hand, can aid the entry of larger molecules into the nucleus by modifying them with a nuclear localization signal, which is an amino acid sequence facilitating transit through the nucleus transport, regardless of the size of the NPCs. Additionally, nanoparticles pass through NPCs via Brownian motion, while NLS modification allows them to specifically target transporters on the nucleus. One well-studied NLS is SV40, which is derived from a tumor antigen of the simian virus 40. Phelippe et al. [101] investigated the process of SV40-conjugated complexes crossing NPCs, involving two steps: binding and translocation. The binding process does not require ATP, but translocation is ATP-dependent and occurs through the importin α/β pathway. Another widely used NLS in nuclear-targeting strategies is the TAT peptide derived from the human immunodeficiency virus type 1 (HIV-1). Pan et al. [102] developed mesoporous silica nanoparticles conjugated with the TAT peptide. With TAT modification, the system successfully translocated a 50 nm complex to the nucleus, which is much larger than the NPCs’ diameters, enhancing the efficiency of DOX loaded in the complex. Various materials have been used to prepare nanoparticles conjugated with TAT, and the complex size can reach up to 50 nm [103,104,105,106]. The drug–NLS complex also possesses a high capability of enhancing cell and nucleus uptake rates due to its high positive charge contributed by plenty of lysine and arginine, which promotes strong binding to membranes [96].

### 6.3. Nucleus as a Target for Anticancer Treatment

Since the dysfunction of the nucleus would cause genetic disorders, such as cancer, the cell nucleus is an ideal target for efficient anticancer treatment by damaging DNA or inhibiting the enzymes involved in DNA replication. Many developed drugs, such as doxorubicin, cisplatin, topotecan, camptothecin and gemcitabine, can bind to DNA to damage its structures, inhibit DNA topoisomerase activities, or interrupt DNA chain elongation by competing with deoxycytidine triphosphate.

However, drug mis-delivery may occur due to various reasons including lysosomal sequestration, acidification of the cytoplasm, and drug exclusion effects [107,108], leading to drug resistance in cancer cells. For example, Tada et al. [109] demonstrated that the expression of efflux pump proteins MDR1, MRP1, MRP2 and MRP3 was higher in bladder tumors after anticancer treatment. Beretta et al. [110] also reveal mechanisms of resistance to camptothecin including mis-location in other organelles and induction of ABC transporters. These challenges in drug delivery can significantly impact the effectiveness of therapies aimed at the nucleus, emphasizing the need for nucleus-targeted delivery systems for further research and innovative approaches to overcome drug resistance and enhance the precision of cancer treatments.

## 7. Other Structures in Cells

### 7.1. Golgi Apparatus

There are other cellular structures that play significant roles either in drug distribution or as therapeutic targets. The Golgi apparatus (GA), which follows the ER in the protein processing pathway, is involved in various progresses that contribute to the malignant phenotype of cancer cells. These include modifying and trafficking secreted proteins, regulating extracellular matrix composition, and facilitating glycosylation processes that alter cancer cell adhesion, migration, and evasion [111]. Consequently, a diverse range of potential drugs targeting the GA has been developed, exploring different mechanisms for cancer treatment.

Monensin, an ionophore that mediates the influx of Na^+^ and the efflux of H^+^, disrupts cellular ion balance, thereby impacting vesicle transport, glycosylation, and triggering cell apoptosis [112,113]. Chen et al. [114] developed hybrid erythrocyte and tumor cell membrane-coated nanoparticles (Hyb-NP) to deliver monensin to the GA. Monensin, in turn, inhibited the exocytosis of Hyb-NP from the GA, efficiently preventing metastasis initiation. Additionally, many derivatives of Brefeldin A, SecinH3, and AMF-26 have been developed to inhibit ADP-ribosylation factors (Arfs), key proteins located on GA that regulate membrane trafficking and actin remodeling, impacting tumor migration, invasion, and metastasis [115]. On the other hand, the GA can sequester compounds, causing drug resistance by reducing the concentration of active drugs at their target sites. Krise et al. [116] demonstrated that the zwitterionic compound Sulforhodamime 101 can be sequestered into the GA through a drug transporter-mediated process, leading to drug resistance by reducing the concentration at active sites. A study by Merlin et al. [117] showed that the drug resistance of daunorubicin can be overcome by using P-gp blockers like S9788, which reduce drug trapping in the GA without altering efficacy.

### 7.2. Endosomes

For drugs that require distribution via delivery systems, such as DNA, RNA, proteins, and peptides, endocytosis often leads to their sequestration within endosomes, limiting their intracellular distribution and reducing therapeutic efficiency [118,119]. The conjugation of targeting domains, such as N-acetylgalactosamine (GalNAc), which binds to the asialoglycoprotein receptor (ASGPR) highly expressed liver hepatocytes, dramatically increases the uptake of RNA therapeutics into endosomes. However, <1% of endocytosed GalNAc-conjugated siRNA escapes from endosomes in vivo [120]. Similarly, a study by He et al. [121] demonstrated that only 1–2% of GalNAc-conjugated antisense oligonucleotides (ASO) escape endosomes in vivo.

To overcome endosomal entrapment, various strategies have been developed. Akita et al. [122] designed a multi-layered nanoparticle system that can fuse with intracellular membranes and release its contents. Polycations like polyethylenemine (PEI) are used as carriers due to their ability to escape from membrane structures by causing membrane destabilization and increasing permeability, thus enhancing the cargo release [123]. Small-molecule endolytics such as Retro-1, guanabenz, and UNC7938 [124,125,126] have been shown to enhance the endosomal escape of RNA therapeutics, but they are also associated with cytotoxicity concerns. As these endolytic agents cause the endosome to burst, achieving significant escape without including cell death or toxicities remains challenging.

Inefficient endosomal escape is the primary barrier to the broad application of RNA therapeutics. Genetic knockdown screens have yet to identify targetable proteins that significantly enhance oligonucleotide activity. Efforts to enhance siRNA activity through molecules that increase endosomal permeability have been limited by toxicity issues. Therefore, novel approaches are needed to improve the cytosolic delivery of oligonucleotides without disrupting endocytic compartments, to address the delivery challenges limiting the therapeutic use of these molecules.

### 7.3. Exosomes

Exosomes, naturally occurring small membrane vesicles (30–150 nm in diameter), are secreted by cells. They are formed through the inward budding of the endosomal lumen layer and carry cellular components, including lipids, proteins, and nucleic acids. Serving as vectors for intercellular communication, the generation levels and contents of exosomes dynamically change, mediated by the cell status and the surrounding microenvironment [127].

Cancer cells tend to produce higher levels of exosomes under stress compared to normal cells. Studies by Wang et al. [128,129] have demonstrated that cytotoxics such as DOX and paclitaxel stimulate the production and release of exosomes containing high drug levels, allowing cells to reduce drug concentration through the efflux of intracellular drug content. Furthermore, co-treatment with exosome inhibitors (GW4869 and omeprazole) significantly restores drug effects by reducing the formation and release of exosomes.

Exosomes also play a role in mediating drug resistance in drug-sensitivity cells by transferring efflux pumps and functional proteins/miRNAs from drug-resistant cancer cells, which promote cancer cell proliferation and survival [130]. Due to the role of exosomes as vehicles for delivering various contents to cancer cells, they are efficient therapeutic targets by incorporating anti-cancer drugs. Unlike liposomes and other synthetic nanoparticle carriers, exosomes contain transmembrane and membrane-anchored proteins that may enhance endocytosis. Kamerkar et al. [131] engineered exosomes to deliver interfering RNA to specifically target oncogenic KRAS in pancreatic cancer, which can suppress cancer growth by enhancing macropinocytosis.

### 7.4. Liquid–Liquid Phase Separation

Within cells, there are membrane-less structures formed based on the liquid–liquid phase separation (LLPS) effect [1,132,133]. These structures can concentrate different complexes and promote bioreactions. The study conducted by Kojima et al. [134] revealed a nanoparticle (NR-PSt NPs) uptake into LLPS droplets and the results show the complex of LLPS and NP has high stability. The Aberrant LLPS could be a cause of cancer due to abnormal biomolecular condensates and assembly of membrane-less organelles [135]. Takayama et al. [136] demonstrated LLPS formation induced by transcription factors contributes to cancer aggressiveness and drug resistance, and the inhibition of it can overcome drug resistance. Drugs can also accumulate in LLPS, but the impact of LLPS on drug effectiveness is not yet fully understood [137]. The mediator complex subunit 1 (MED1) is a key co-activator protein that is a defining component of transcriptional condensates. One study showed that LLPS formed by MED1 droplets can concentrate cisplatin in it and enhance anticancer efficacy [138].

**Figure 1:**
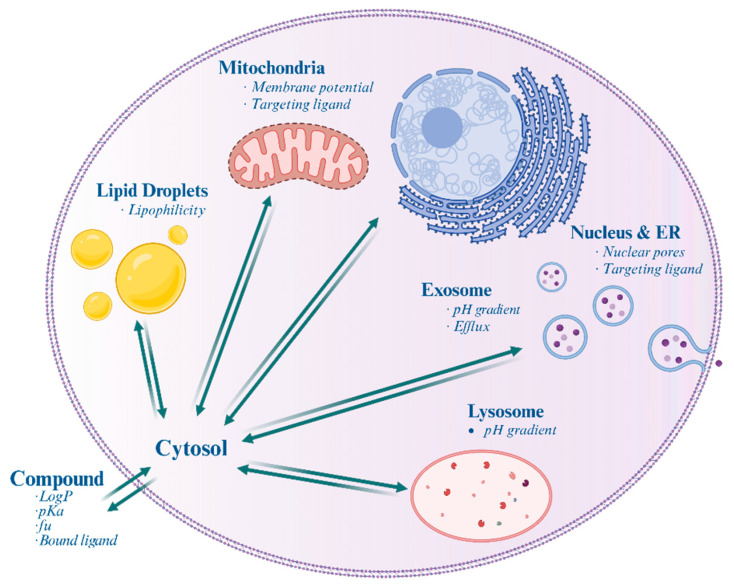
Schematic illustration of a mammalian cell highlighting the differential accumulation of drugs within various subcellular organelles and structures. Italics indicate the factors affecting accumulation.

Overall, understanding the involvement of various cellular structures (Figure 1, Table 1) in drug distribution and resistance is essential for developing effective therapeutic strategies in cancer treatment and other diseases.

## 8. Subcellular Drug Distribution Models

The subcellular distribution of drugs plays a crucial role in their effectiveness, and alterations in distribution are often implicated in drug resistance mechanisms. To better understand and predict the distribution of various drugs at the organelle levels, computational modeling approaches based on the physicochemical properties of drugs have emerged as potentially useful tools for estimating the concentrations in subcellular compartments. An ideal model for accurately predicting drug distribution at the subcellular level relies on precise physicochemical property data and requires validation with accurate experimental datasets that provide quantitative measurements of drug concentrations in specific subcellular compartments. In these computational models, experimental or in-silico simulated properties of drugs, such as their chemical nature (acidic, basic, neutral), physicochemical properties (logP, pKa, permeability value of the membrane for ionized molecules), protein binding affinity, etc. are preset as parameters. Mechanism-based physiological models are then constructed, and regression analysis methods are applied to train the model using experimental datasets to verify its fitting effectiveness. Subsequently, these trained models can be utilized to predict the localization of drugs within the cell [139,140,141]. By leveraging computational modeling, researchers can gain valuable insights into drug distribution patterns, aiding in the design of more effective therapeutic strategies and potentially identifying factors contributing to drug resistance.

### 8.1. Static Model of Predicting Steady-State Subcellular Drug Distribution

In physiologically based pharmacokinetic (PBPK) models, the tissue partition coefficient (Kp) is the most central parameter that describes the concentration ratio between plasma and tissue. This ratio is influenced by various factors, such as drug binding with proteins/lipids, drug dissociation state under specific pH conditions, membrane potential between sub-compartments, and the expression level of transporters that the drug is subjected to, among others [142]. Rodgers et al. [143,144] developed a model based on drug properties and cell compositions for the prediction of Kp. The drugs were classified into several groups: Moderate-to-Strong basic drugs, and drugs of Acids, Very Weak Bases, Neutrals, and Zwitterions. Compounds ionized in the cellular microenvironment, and only neutral molecules were allowed to pass through membranes. Tissues were no longer treated as a well-stirred compartment; instead, extracellular space, intracellular space, and different phospholipids to which drugs bind were considered sub-compartments. The concentrations of compartments were predicted based on drug LogP and pKa values, and the total tissue concentration was calculated based on the concentrations of each sub-compartment. This tissue-composition-based model can generate different Kp values depending on the compositions of phospholipids present in different tissues.

Building on this work, Assmus et al. [145] created an improved model that included lysosomes as a sub-compartment of the cell. For basic drugs, this model accounted for their sequestration in lysosomes due to pH gradients. It was able to predict the lysosome concentration and calculate Kp for different tissues based on their lysosome volume fraction and cell compositions. This model was applied in the GastroPlus^TM^ PBPK Simulator program (Simulations Plus Inc., Lancaster, CA, USA). Liu et al. [146] extended the PBPK model to include lysosome sub-compartments and applied the same concepts to describe the pharmacokinetic profile of chloroquine in rats and humans at both tissue and organelle levels. They explained the shift in tissue Kp values over time by considering changes in lysosome pH. Since lysosome pH is a critical factor in predicting lysosome concentration, the accumulation of certain compounds can alter lysosome pH and subsequently affect the subcellular distribution of drugs. Ishizaki et al. [147] demonstrated that drug accumulation in lysosomes can be inhibited through basic compounds raising the lysosome pH, and successfully established a lysosomal distribution model to simulate this process. Ouar et al. [148] also studied that raising lysosomal pH by inhibiting H^+^-ATPase can reverse drug resistance to anthracyclines in renal cancer. Building on the concept applied in these models (Table 2), organelles are treated as sub-compartments of cells and the concentration corresponding to them can be predicted based on drug characteristics and composition of organelles.

### 8.2. Dynamic Models with the Nernst-Plank Equation

The models discussed above are capable of predicting subcellular/cellular level concentrations, but they are limited to simulating only steady-state conditions, not providing insights into the distribution process and equilibrium. Moreover, these models do not account for the diffusion of ionized molecules through membranes. To address these limitations and improve the models, the Nernst–Planck equation can be incorporated. Trapp et al. [152,153,154], developed models using the Fick–Nernst–Planck equation to describe the distribution of both neutral and ionized compounds within specific organelles that possess pH gradients or membrane potentials (e.g., lysosomes or mitochondria). They explored the relationship between drug accumulation and pharmacokinetic properties of drugs. In a study by Collins et al. [155], a model was developed to simulate combination treatments that alter the pharmacokinetic profile of hydroxychloroquine (HCQ) in cancer. This model investigated the role of lysosomes in HCQ pharmacokinetics and predicted tissue concentrations in mice.

Physicochemical-based models can be used to guide the design and optimization of organelle-targeted agents. These models incorporate the metabolic characteristics of drugs within the body, allowing for the prediction of drug distribution as well as the description of their transfer and elimination at the subcellular level. Horobin et al. [154] conducted a review of quantitative structure–activity relations (QSAR) models for mitochondria-specific compounds and provided guidelines for optimizing mitochondriotropic characteristics. Zhang et al. [156] developed a cell-based molecular transport simulator to explore chemicals for chemical genomics and drug discovery. The incorporation of the Nernst–Planck equation and other physicochemical considerations enhances the modeling capabilities, providing a more comprehensive understanding of drug distribution and facilitating the development of targeted agents for specific organelles. In addition to the mechanism-based strategies to mimic the distribution behavior of drugs, neural network models with neural ordinary differential equations are useful complements to describe data profiles that the mechanism was not clearly known [157].

The subcellular distribution of drugs is critical for determining their effectiveness and mechanisms of resistance. Computational models, which depend on precise physicochemical data and accurate experimental datasets, are often limited by the scarcity of quantitative measurements in specific subcellular compartments. Despite advancements, such as incorporating organelle sub-compartments, these models require more robust experimental techniques and comprehensive datasets for validation. Enhanced methods are essential for providing the necessary data. Increased research and resources are needed to refine these models and achieve high-confidence predictions for subcellular drug distribution.

## 9. Methodology for Detecting Subcellular Concentrations

In early cell fractionation studies, organelles and subcellular components were isolated from sample homogenates through differential centrifugation, and the drug concentration was measured in each fraction solution [158,159,160]. This approach relies on the accurate and reliable separation of organelles to map drug distribution at the subcellular level; however, the multistep isolation process can inherently disrupt the drug distribution within organelles.

As interest in chemical analysis at the subcellular level grows, innovative qualitative and quantitative analytical methods are increasingly being developed. Although a definitive gold standard for single-cell or subcellular compound monitoring has not yet been established, these novel techniques can generally be categorized into three major classes widely used in the field.

### 9.1. Imaging-Based Techniques for Subcellular Analysis

Imaging is one of the most commonly used techniques for studying the structure and characteristics of cells and organelles. Besides the intrinsic properties of fluorophores that affect the accuracy and reliability of measurements, signal diffraction is a key limiting factor in determining imaging resolution. To address this limitation, various super-resolution microscopy (SRM) techniques have been rapidly developed (Table 3) [161]. These methods provide high-quality images, allowing researchers to quantitatively explore the subcellular systems. Kensei et al. [162] applied structured illumination microscopy (SIM), which employs patterned light and computational reconstruction to achieve super-resolution, to evaluate the mitochondrial morphology by quantifying mitochondrial volume and surface area. They found that the mitochondrial network associated with this area breaks down with kidney injury. For subcellular distribution studies using SRM, drugs must be labeled with fluorophores or possess intrinsic fluorescence. Chen et al. [163] developed a complex called a drug beacon, conjugated with coumarin–hemicyanine as a luminophore, allowing for direct visualization and quantification of complex distribution using SIM. Magnoflorine, which has autofluorescent properties, was studied by the same group [164], providing a description of its subcellular distribution and mechanistic target using SIM.

Additionally, super-resolution optical fluctuation imaging (SOFI) was used to quantify the distribution of proteins labeled with suitable blinking fluorophores on the cellular surface of T cells [165], demonstrating potential applications for any membrane surface under various conditions. Another method, single-molecule localization microscopy (SMLM), achieves high-resolution images far beyond the diffraction limit by using novel parameter-free algorithms. Marenda et al. [166] utilized this technique to characterize and quantify the superstructure between protein clusters in the nucleus.

SRM significantly enhances the ability to determine molecular locations within cells, surpassing the diffraction limit of conventional microscopy. This technique enables the precise visualization of molecular distributions and interactions at the nanometer scale. However, there are notable limitations [167]. These techniques often require specialized equipment and complex sample preparation, which can restrict their use. The necessity for fluorescent dyes or labels may not be applicable to all drugs and could alter their behavior. Additionally, SRM can be time-consuming and involve complex data processing, potentially introducing artifacts.

### 9.2. Electrochemical Analysis Techniques

Electrochemical analysis is another widely used technique for monitoring the intricate dynamics of drug distribution and interaction within cells. Utilizing various types of electrodes, these methods provide insights into the quantification of vesicular molecule storage and release, significantly enhancing our understanding of drug distribution and efficacy at the subcellular level.

Single-cell amperometry (SCA) is one such technology that quantifies the number of molecules released from cells. For example, Tang et al. [168] used a nanoelectrode to quantify dopamine release inside single synapses, demonstrating that harpagide can restore dopamine release levels in a Parkinson‘s disease model. Intracellular vesicle impact electrochemical cytometry (IVIEC) is another technique used to analyze vesicular contents within cells. Roberts et al. [169] used a carbon-fiber nanometer-scale electrode inserted into individual neuroendocrine cells to distinguish vesicles containing epinephrine from those containing catecholamine. By combining SCA and IVIEC, Taleat et al. [170] measured both exocytosis and intracellular catecholamine levels, revealing that the anticancer drug tamoxifen inhibits catecholamine release at high concentrations and stimulates it at low concentrations, potentially leading to memory and cognitive dysfunction. Electrochemical measurements have also been applied to monitor reactive oxygen and nitrogen species (ROS/RNS), and nicotinamide adenine dinucleotide (NADH) [171,172]. These applications further demonstrate the versatility and importance of electrochemical techniques in studying subcellular processes and drug interactions.

From another perspective, these methods usually require precise electrode placement and calibration, which can be challenging and may not suit all cell types. And they also offer limited spatial resolution compared to imaging techniques and demand specialized electrodes and expertise, limiting their broader application. Moreover, while these methods are excellent for quantifying specific molecules, they may not fully capture the broader context of drug behavior across various cellular compartments [173,174].

### 9.3. Advanced Mass Spectrometry Methods for Single-Cell and Subcellular Analysis

In recent years, advancements in mass spectrometry have enabled the analysis of very small volumes within signal cells [175]. Coupled with microcapillary sampling tips, nano-ESI-MS can directly detect small molecules in a live, single cell and its organelle. Mizuno et al. [176] combined this technique with video-microscopy. When a specific response or organelle is observed, the contents of these micro-regions are sampled using an ionization tip, allowing the identification and quantification of molecules corresponding to MS peaks. Zhao et al. [177] developed in-tip solvent microextraction MS for profiling lipids within a single lipid droplet, distinguishing phosphatidylcholines (PCs) and triglycerides (TGs). Another derivative of MS is based on laser-desorption/ionization (LDI), allowing samples to be irradiated by a laser beam before MS analysis. By accurately extracting a known volume of cellular content from a single plant cell, Zhao et al. [178] applied hydrogen flame desorption ionization MS (HFDI-MS) to quantify analytes of interest at the picoliter level without needing an internal standard.

Mass spectrometry is also a high-throughput approach for single-cell analysis. Neumann et al. [179] adapted matrix-assisted LDI-MS to profile lipid composition in mammalian cells, analyzing 30,000 cells and detecting 520 lipid features. Secondary ion (SI) and inductively coupled plasma (ICP) mass spectrometry are also adapted to single-cell analysis [180,181,182], demonstrating the critical role of MS in subcellular molecule distribution analysis.

Like other techniques, these methods also have limitations [183]. They often require intricate sample preparation and specialization. Despite their high sensitivity and resolution, these techniques may not always capture the full spatial context of drug distribution within cells. Additionally, their varying sensitivities can impact the detection of low-abundance analytes and the accuracy of drug distribution measurements. Moreover, the high-throughput nature of mass spectrometry can pose challenges in data interpretation, necessitating advanced computational and bioinformatic analyses.

**Table 3:** Method for single-cell/subcellular drug determination.

Category	Techniques	Advantages	Limitations
Super-resolution microscopy Method	Expansion microscopy [184] Image scanning microscopy [185] Super-resolution optical fluctuation imaging [186] Stimulated emission depletion microscopy [187] Single-molecule localization-based SRM [188]	High Spatial Resolution Dynamic Imaging Single-Molecule Sensitivity	Complex Sample Preparation Phototoxicity and Photobleaching
Electrochemical Method	Single-Cell Amperometry [189] Intracellular Vesicle Impact Electrochemical Cytometry [169] Enzymatic Biosensors [190]	High Sensitivity High Specificity Rapid Response	Electrode Invasiveness Complex Calibration Electrode limitation
Mass spectrometry Method	Nano-Electrospray ionization-MS [177] Laser-desorption/ionization-MS [191] Secondary ion-MS [192] Inductively coupled plasma-based-MS [193]	High Sensitivity and Specificity Wide range applicability High Throughput	Sample destruction Expertise Required Poor spatial resolution

## 10. Conclusions

To enhance the effectiveness of drugs, it is essential to deliver therapeutic molecules not only to the appropriate tissue-level site but also to the precise subcellular location where the drug can reach an effective concentration without causing potential side effects. Engineering organelle-targeted modifications of drugs is a popular and feasible strategy, as organelles play critical roles in biological processes and possess specific characteristics that can be exploited. For instance, modifying drugs to target specific organelles allows the payload to reach active locations and achieve the desired therapeutic effects.

Conversely, cells, especially cancer cells, may adopt similar strategies in the opposite direction to survive under treatment conditions. They can alter the distribution of drugs to reduce the concentration in active locations and actively efflux the molecules extracellularly, leading to drug resistance. For example, drugs trapped in specific organelles that are not sensitive to the drugs can confer drug resistance to targeted cells, such as lysosome-trapped chloroquine impacting drug efficiency [153] or lipid droplet accumulation reducing the free fraction concentration of compounds in the intracellular space [194]. Additionally, overexpression of plasma membrane drug efflux pump proteins like P-gp and MRP can contribute to altering drug distribution and reducing effective drug levels. Overcoming alterations in drug distribution in drug-resistant cells and directing drugs to their desired subcellular locations remains a challenging task for further research.

Computational pharmacokinetic models are powerful tools for predicting drug behavior and have been widely used to describe organ-level drug disposition. They have also been applied to establish intracellular models. However, subcellular models have their limitations, such as data uncertainties, feedback mechanisms occurring during distribution processes, influences of associated transports, and the accuracy of physical and chemical parameters. Creating subcellular distribution models requires more complex model structures and more accurate parameter values to predict distribution among various organelles and cellular components. Therefore, innovative analytical methods with high sensitivity and spatial–temporal resolution that allow qualitative and quantitative analysis are gaining increased attention for use at the subcellular level. The development of new techniques in this area can provide a stronger foundation for subcellular models.

## Figures and Tables

**Table 1:** Key Organelle Features Dictating Subcellular Drug Distribution Patterns.

Organelle	Key Features Affecting Drug Distribution	Impact on Subcellular Drug Distribution/Efficacy	Drugs or Ligands Accumulating
Lysosomes	Acidic pH gradient	Lysosomal trapping reduces cytoplasmic drug levels	Sunitinib, Lapatinib, Gefitinib [2,6]
Mitochondria	Lipophilic cations Negative membrane potential Mitochondrial targeting peptides	Targeting mitochondria can enhance efficacy in mitochondrial disorders	Mitochondrial-penetrating peptide (MPP) [42], Triphenylphosphonium (TPP) [37]
Lipid Droplets	High lipophilicity	Sequestration in lipid droplets reduces cytoplasmic drug levels	Gefitinib [82], Lasonolide A [87]
Nucleus	Size/shape restrictions of nuclear pores Nuclear localization signals (NLS)	Targeted delivery raises drug levels in nucleus	TAT peptide [103,104,105,106]
Other Structures	Targeting peptides pH gradient Ion strength	ER/Golgi trapping can enhance drug efficacy Endosomal entrapment and exosomal efflux of drugs reduce cytoplasmic drug levels	Sulforhodamime 101 (golgi) [116]; KDEL peptide (ER);

**Table 2:** Computational Approaches for Modeling Subcellular Drug Distribution.

Model	Main Assumptions/Features	Key Parameters	Advantage(s)
Poulin method [149,150]	passive diffusion Tissues consist of water, lipids and proteins	logP, *fu* (fraction unbound)	Simple structure
Berezhkovskiy method [151]	Ibid. Drug binding to lipids does not bind to proteins	logP, *fu*	Simple structure; Restrict only free drug binding to protein/lipid
Rodgers and Rowland method [143,144]	Ibid. Ionized basic drugs bind to acidic phospholipids Classify drugs according to pKa	logP, *fu*, pKa, B/P ratios	Accurate description of binding to different compositions (proteins/lipids); Allow calculating concentration of organelles
Lukacova method	Uniform equation applied to drugs with different pKa Extended with organelle compartments	logP, *fu*, pKa, B/P ratios	Simplified equations for applying to all types of drugs

## Data Availability

Not applicable.
